# Hypotony Management through Transconjunctival Scleral Flap Resuturing: Analysis of Surgical Outcomes and Success Predictors

**DOI:** 10.5005/jp-journals-10028-1224

**Published:** 2017-08-05

**Authors:** Ana Luiza B Scoralick, Izabela Almeida, Michele Ushida, Diego T Dias, Syril Dorairaj, Tiago S Prata, Fábio N Kanadani

**Affiliations:** 1 Staff specialist, Department of Ophthalmology, Instituto de Olhos Ciencias Medicas, Belo Horizonte, Brazil; 2 Postgraduate Student, Department of Ophthalmology, Glaucoma Service, Federal University of Sao Paulo, Brazil; Glaucoma Unit Hospital Medicina dos Olhos, Osasco, Brazil; 3 Staff specialist, Glaucoma Unit, Hospital Medicina dos Olhos, Osasco, Brazil; 4 Postgraduate Student, Department of Ophthalmology, Glaucoma Service, Federal University of Sao Paulo, Brazil; Glaucoma Unit Hospital Medicina dos Olhos, Osasco, Brazil; 5 Assistant Professor, Department of Ophthalmology, Mayo Clinic, Jacksonville Florida, USA; 6 Associate Professor, Department of Ophthalmology, Glaucoma Service, Federal University of Sao Paulo, Brazil; Glaucoma Unit Hospital Medicina dos Olhos, Osasco, Brazil; Department of Ophthalmology Glaucoma Service, Sorocaba Ophthalmology Hospital, BOS Sorocaba, Brazil; 7 Associate Professor, Department of Ophthalmology, Instituto de Olhos Ciencias Medicas, Belo Horizonte, Brazil

**Keywords:** Choroidal detachment, Glaucoma, Hypotony maculopathy, Scleral flap resuturing, Trabeculectomy, Transconjunctival.

## Abstract

**Aim:**

To investigate surgical outcomes and success predictors of transconjunctival scleral flap resuturing for the management of hypotony due to overfiltration following trabeculectomy with mitomycin C.

**Materials and methods:**

Noncomparative, retrospective, interventional case series in which all glaucoma patients from two glaucoma services undergoing transconjunctival scleral flap resuturing between May 2012 and July 2016 were enrolled. Included eyes had to have hypotony [intraocular pressure (IOP) < 6 mm Hg] and/or hypotony maculopathy caused by excessive filtration following trabeculectomy. Key exclusion criteria were wound/bleb leaking and postoperative ocular trauma or infection. Preoperative and postoperative IOP, best-corrected visual acuity (BCVA), fundus imaging, surgical complications, and any subsequent related events or procedures were recorded. Rates of postsurgical hypotony and/or maculopathy resolution and possible success predictors were investigated.

**Results:**

A total of 22 patients (22 eyes) with a mean age of 56.4 ± 15.2 years were included. Median follow-up was 245 days [interquartilerange (IR); 120-817 days] and mean IOP was increased from 2.9 ± 1.5 mm Hg (1-6 mm Hg) to 8.5 ± 3.1 mm Hg (2-16 mm Hg) at the last follow-up visit (p < 0.01). Approximately 75% of the cases (16 out of 22) had an IOP between 7 and 18 mm Hg at the end of the follow-up period. Median BCVA (log MAR) at last follow-up visit [0.1 (IR; 0.0- 0.3)] was significantly better than preoperative BCVA [0.4 (IR; 0.11.0); p < 0.01]. Hypotony resolved in 81% of the cases, while maculopathy resolution was found in 85% of the cases. Time interval between trabeculectomy and flap resuturing was the only factor significantly associated with patient’s IOP at last follow-up visit (R^2^ = 0.23; p = 0.036). Success rates (IOP > 6 mm Hg at last follow-up visit) were halved in those left untreated for more than 6 months. No serious adverse event was recorded.

**Conclusion:**

Our findings support the use of transconjunctival scleral flap resuturing as an effective and safe alternative for hypotony management due to overflitration following trabeculectomy. As time interval seems to influence the odds of hypotony resolution, early intervention is recommended.

**How to cite this article:**

Scoralick ALB, Almeida I, Ushida M, Dias DT, Dorairaj S, Prata TS, Kanadani FN, Hypotony Management through Transconjunctival Scleral Flap Resuturing: Analysis of Surgical Outcomes and Success Predictors. J Curr Glaucoma Pract 2017;11(2):58-62.

## INTRODUCTION

Since its introduction 40 years ago, trabeculectomy has become the most frequently performed surgical procedure for glaucoma.^1^ The adjunctive use of antifibrotic agents, such as mitomycin C (MMC) in filtration surgery, has increased success rates, but also led to a higher incidence of ocular hypotony.^2,3^ Low intraocular pressure (IOP) attributable to overfiltration following trabecu-lectomy is a common early postoperative complication, ranging from 1 to 18% of the cases.^2-4^ It may lead to flat anterior chamber,^5^ choroidal detachment,^6^ hypotony maculopathy,^7^ optic disk edema,^8^ cataract development,^9^ and reduced visual acuity.^10^

The way each individual eye is affected by low IOP values may vary significantly. In this context, hypotony definition itself may also vary. When we think about objective absolute values, hypotony has been defined as an IOP less than 6.5 mm Hg by Pederson.^11^ Considering a more clinical point of view, hypotony has been considered as a condition in which IOP is low enough to result in anatomical and/or functional disturbances.^12^ It should be emphasized that hypotony may be accompanied by subclinical maculopathy in some cases, in which macular changes are only detected by high-resolution imaging devices.^13^ If not treated in a timely manner, hypotony maculopathy may result in permanent structural and functional damage.^10,14^

Many techniques have been developed to treat over-filtration following filtering procedures, such as bandage contact lens,^15^ intrableb autologous blood injection,^16^ cryotherapy,^17^ laser therapy (argon, Nd:YAG),^18^ compression mattress sutures,^19-21^ combined conjunctival compression sutures and autologous blood injection,^19,20^ and surgical revision of the trabeculectomy (open site flap resutur-ing).^22^ Overall, outcomes seem to vary significantly between studies and according to each case, and none of the above-mentioned methods has been adopted as a gold standard option for successfully managing such cases.^23,24^ In addition, these surgical interventions may also be associated with complications, such as hyphema, decreased vision, trabeculectomy failure, pain, and infec-tion.^25^ In this study, we sought to investigate surgical outcomes and success predictors of transconjunctival scleral flap resuturing - a minimally invasive not so widespread technique - for the management of hypotony due to overfiltration following trabeculectomy with MMC.

## MATERIALS AND METHODS

### Patients

A noncomparative, retrospective, interventional case series was carried out. All glaucoma patients from two glaucoma services (Instituto de Olhos Ciencias Medicas and Hospital Medicina dos Olhos) undergoing transconjunctival scleral flap resuturing between May 2012 and July 2016 were enrolled. Included eyes had to have hypotony (IOP < 6 mm Hg) and/or hypotony maculopathy caused by excessive filtration following trabeculectomy. Exclusion criteria were wound/bleb leaking, postoperative ocular trauma or infection. Preoperative and postoperative IOP, best-corrected visual acuity (BCVA; log MAR), fundus imaging, surgical complications, and any subsequent related events or procedures were recorded. Rates of postsurgical hypotony and/or hypotony maculopathy resolution and possible success predictors were investigated.

### Procedure

Topical anesthesia was administered using xylocaine gel 2%. Topical 5% povidone-iodine solution eye drops were then instilled. A lid speculum was used to facilitate the procedure and to limit the risk of wound contamination by lashes. Through the intact conjunctiva, a minimum of two 10.0 nylon sutures were placed through the scleral flap, then passed through the adjacent sclera and conjunctiva, and knotted tightly over. All sutures were then buried. Postoperatively, all patients were treated with local antibiotics and steroids, tapered along 14 days.

### Statistical Analysis

Descriptive analysis was used to present demographic and clinical data. D’Agostino-Pearson’s test was performed to determine whether the data had a normal distribution or not. Descriptive statistics included mean and standard deviation for normally distributed variables and median, quartiles for those non-normally distributed. Paired samples t-test was used to compare continuous normally distributed variables before and after flap resuturing, whereas the Wilcoxon test was used to compare those non-normally distributed. Scatter plots and regression lines were constructed to investigate possible factors associated with patient’s IOP at last follow-up visit (final IOP after flap resuturing; considered as a continuous variable). Logistic regression analysis was used to investigate possible factors associated with maculopathy resolution following flap resuturing (adjusting for IOP changes following flap resuturing). Computerized analysis was performed using MedCalc software (MedCalc Inc., Mariakerke, Belgium). The alpha level (type I error) was set at 0.05.

## RESULTS

A total of 22 eyes from 22 patients with a mean age of 56.4 ± 15.2 years ( 25-74) were included. Patient’s demographic characteristics are summarized in Table 1. Median follow-up was 245 days [interquartilerange (IR); 120-817 days) and mean IOP was increased from 2.9 ± 1.5 mm Hg (1-6 mm Hg) to 8.5 ± 3.1 mm Hg (2-16 mm Hg) at the last follow-up visit (p < 0.01; Table 2). Approximately 75% of the cases (16 out of 22) had an IOP between 7 and 18 mm Hg at the end of the follow-up period. Median BCVA (log MAR) at last follow-up visit [0.1 (IR; 0.0-0.3)] was significantly better than preoperative BCVA [0.4 (IR; 0.1-1.0); p < 0.01].

**Table 1:** **Table 1:** Demographic characteristics of study patients

Variables *			
Age (years)		56.4 ± 15.2	
Gender % (F/M)		59/41	
Race % (W/AD/O)		54/14/32	
Glaucoma type % (POAG, O)		91/9	

M: Male; F: Female; W: White; AD: African descending; O: Others; POAG: Primary open angle glaucoma

*Data are given as mean ± standard deviation whenever indicated

**Graph 1:**
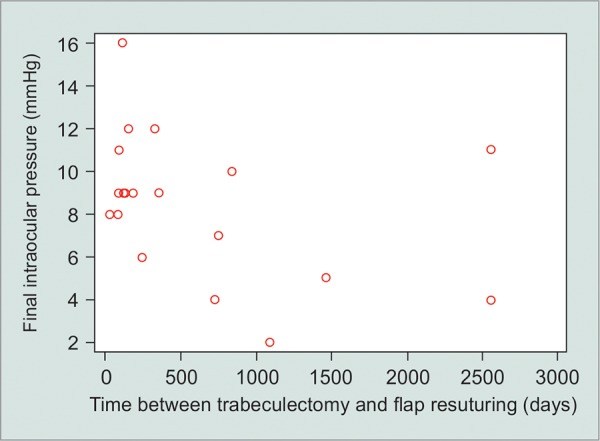
Scatter plot of time interval between trabeculectomy and the flap resuturing (days) against intraocular pressure values at last follow-up visit

Ocular hypotony resolved in 81% of the cases (eyes with IOP > 6 mm Hg at last follow-up visit), while macu-lopathy resolution was found in 85% of the cases. Time interval between trabeculectomy and flap resuturing was the only factor significantly associated with patient’s IOP at last follow-up visit (R^2^ = 0.23; p = 0.036; Graph 1); success rates (IOP > 6 mm Hg at last follow-up visit) were halved in those left untreated for more than 6 months. None of the variables investigated (age, time interval between trabeculectomy and flap resuturing, race, type of glaucoma, and baseline BCVA; adjusting for IOP changes following flap resuturing) were significantly associated with postoperative maculopathy resolution (p > 0.05).

**Table 2:** **Table 2:** Clinical outcomes after transconjuntival resuturing

Variables*		*Before suturing*		*After suturing*		*p-value*	
IOP		2.9 ± 1.5		8.4 ± 3.2		<0.001	
BVCA		0.4 (0.1, 1.0)		0.1 (0.0, 0.3)		<0.001	
Hypotony maculopathy		91%		15%		<0.001	

Ivariables represented by median (first quartile, third quartile)

One patient presented with choroidal detachment (IOP = 4 mm Hg) at initial visit (following trabeculec-tomy). He was treated with transconjunctival scleral flap resuturing combined with choroidal drainage. After surgery, choroidal detachment was not observed and IOP increased to 8 mm Hg.

**Figs 1A and B:**
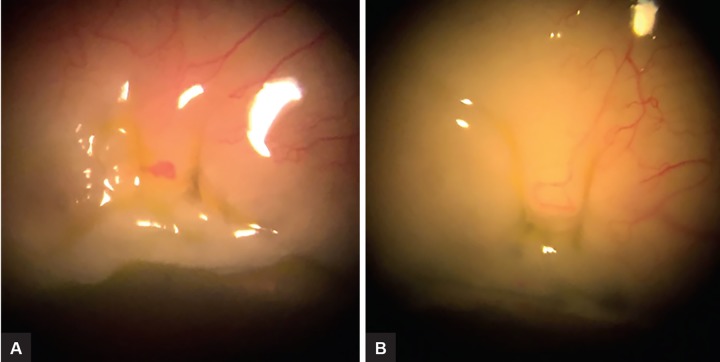
Transconjunctival scleral flap resuturing: (A) Two sutures were placed; and (B) two days after the procedure, sutures were already buried under the conjunctiva

No serious adverse event was recorded. In the majority of the cases, sutures were already buried under the conjunctiva after only a few days after the procedure (Figs 1A and B). No bleb or wound leakage was noted postoperatively.

## DISCUSSION

Although low IOP attributable to overfiltration following trabeculectomy is a uncommon early postoperative complication, it may lead to serious visual disability due to choroidal effusion, maculopathy, flat anterior chamber, optic neuropathy, and cataract development.^10,23,26,27^ Adjunctive use of antifibrotic agents with trabeculectomy may increase the incidence of postoperative hypotony.^2,3^ Many techniques have been developed to treat overfiltration with hypotony maculopathy following filtering procedures, including transconjunctival scleral flap resu-turing. Although good results (few studies) have been previously reported, this technique does not resolve all cases of hypotony due to overfiltration following trabecu-lectomy. In this context, the study not only reinforces the efficacy and safety data of transconjunctival scleral flap resuturing, but also underlines the time interval between trabeculectomy and flap resuturing as a significant predictor of hypotony resolution. These findings highlight the importance of early surgical reintervention in these cases, as odds of reaching hypotony resolution were halved in eyes left untreated for more than 6 months.

There are scant data in the literature when it comes to transconjunctival scleral flap resuturing. Regarding the few previously published studies, Maruyama and Shirato^23^ described a retrospective study in which mean IOP was 2.9 ± 1.4 mm Hg before and 8.4 ± 4.1 mm Hg at 12 months after flap resuturing. Another retrospective study showed statistically significant (p < 0.01) IOP increase (3-9 mm Hg) and BCVA improvement (20/100-20/30) 6 months after transconjunctival scleral resuturing flap.^25^ Eha et al,^28^ in 2008, published a prospective case study describing the outcome of 16 patients whose mean IOP was 9.6 mm Hg, and mean BCVA was 20/60, 6 months following the procedure. Finally, in 2013, the results of another retrospective study also demonstrated statistically significant IOP increase and BCVA improvement with the same surgical technique.^29^ All these above-mentioned studies focused specifically on procedure’s efficacy and safety, not evaluating factors possibly associated with surgical outcomes. Nevertheless, we believe the results of this study corroborate their findings, as over 80% of our cases presented hypotony and maculopathy resolution, with a very similar mean IOP (8.5 mm Hg) at last follow-up visit.

At this point we believe it is important to discuss the clinical outcomes of the other available alternatives for post-trabeculetomy overfiltration management. Lynch et al.^18^ described a study with 23 patients that underwent YAG laser bleb remodeling due to overfiltration and hypotony. As a result, 64% of the treated eyes had an IOP increase of at least 3.0 mm Hg. Wise^16^ treated four patients with chronic hypotony and decreased vision after filtering surgery with autologous blood injection. In these eyes, mean IOP increased from 5.5 to 8.2 mm Hg following the injection. Nuyts et al,^15^ also performing intrableb autolo-gous blood injection (22 eyes), found a mean IOP increase of approximately 4 mm Hg (from 4.3 ± 1.8 mm Hg before injection to 8.6 ± 4.6 mm Hg after injection). Haynes and Alward^20^ performed a combined procedure of autolo-gous blood injection and bleb compression sutures to treat overfiltration with hypotony maculopathy in two patients and a small IOP increase was reported in both cases (patient 1: 3 to 5 mm Hg; patient 2: 2-5 mm Hg). Palmberg^21^ reported the results of a large series of patients treated with conjunctival compression sutures. Of the 46 treated eyes, 69% were classified as success following the procedure. Quaranta^30^ also performed conjunctival compression sutures for large blebs associated with ocular hypotony after uncomplicated trabeculectomy with MMC. Similar to the previously cited study, 64.4% of the eyes presented hypotony resolution (postoperative mean IOP: 13.4 ± 1.8 mm Hg. It is important to observe that patients profile, success criteria and main endpoints vary between studies, what limits a straight comparison between each technique. What seems clear is that even though IOP increase and visual acuity improvement may be obtained for many patients with such techniques, hypotony may persist in some eyes. Although, success predictors were not investigated in the above mentioned studies, our findings suggested the time interval between trabeculectomy and resuturing of the scleral flap as an important factor for hypotony resolution.

In clinical practice, it is of general consensus that hypotony duration is closely related to maculopathy resolution in eyes with postoperative hypotony. If timely managed, IOP increase usually leads to restoration of the normal smooth architecture of the retina, allowing realignment of photoreceptors and visual recovery.^14,31^ On the contrary, prolonged hypotony may cause irreversible fibrosis within the retina, choroid or sclera, maintaining the choroid in a folded position.^14,31^ In our study, even though maculopathy resolution was found in 85% of the cases following surgical reintervention, none of the variables investigated (including time interval between trabeculectomy and flap resuturing) was significantly associated with postoperative maculopathy resolution. This fact could be explained by the fact that hypotony maculopathy development has multiple underlying predisposing factors.^32^ In this context, two hypotheses arise: (1) None of the factors we investigated has an important effect on maculopathy resolution, (2) the IOP increase itself is strongly correlated with maculopathy resolution, thus mitigating the influence of any other factor.

It is important to stress some specific characteristics and limitations of our study. First, the retrospective (non-comparative) design of our study should be considered while interpreting its results. Second, our study has a limited sample size. A larger number of participants might have uncovered significant relationships that were not observed in our study (thus mitigating the chances of type II error). Third, the association between time interval (between trabeculectomy and flap resuturing) and patient’s IOP at last follow-up visit had a weak coefficient of determination (R^2^ = 0.23). Therefore, this association accounted for only part of the hypotony resolution observed in these patients. Other factors not evaluated in this study might be related to hypotony resolution following transconjunctival sclera flap resuturing in these eyes.

## CONCLUSION

Our findings support the use of transconjunctival scleral flap resuturing as an effective and safe alternative for hypotony management due to overfiltration following trabeculectomy. As time interval seems to negatively influence the odds of hypotony resolution, early reinter-vention is recommended in these cases.
